# First observation of secondary childhood glaucoma in Coffin-Siris syndrome: a case report and literature review

**DOI:** 10.1186/s12886-020-01788-0

**Published:** 2021-01-11

**Authors:** Heidi Diel, Can Ding, Franz Grehn, Panagiotis Chronopoulos, Oliver Bartsch, Esther M. Hoffmann

**Affiliations:** 1 Department of Ophthalmology, University Medical Centre of the Johannes Gutenberg University Mainz, Langenbeckstr. 1, D – 55131 Mainz, Germany; 2 Institute of Human Genetics, University Medical Centre of the Johannes Gutenberg University Mainz, Mainz, Germany

**Keywords:** Coffin-Siris syndrome, Coffin-Siris syndrome 9, *SOX11* gene, Secondary childhood glaucoma, Anterior segment dysgenesis, Peters anomaly, Aniridia, Case report

## Abstract

**Background:**

Severe congenital ophthalmological malformations and glaucoma might be an important occasional feature in patients with Coffin-Siris syndrome (CSS), especially Coffin-Siris syndrome 9 (CSS9, OMIM #615866) caused by *SOX11* mutation. Recently, primary (open-angle) glaucoma was described in two children with the most common form of Coffin-Siris syndrome, CSS1 (OMIM #135900) by *ARID1B* (AT-rich interaction domain-containing protein 1B) gene mutation. In this article, we present the first report of glaucoma with Coffin-Siris syndrome 9 as well as the first report of secondary glaucoma with any form of Coffin-Siris syndrome. These findings indicate that secondary glaucoma is an occasional finding in patients with Coffin-Siris syndrome.

**Case presentation:**

A child with secondary childhood glaucoma and additional ocular manifestations was evaluated and treated at the childhood glaucoma centre in Mainz, Germany. Examination under general anaesthesia revealed ocular anterior segment dysgenesis (ASD) (Peters type iridocorneal dysgenesis) in combination with congenital limbal stem cell deficiency (LSCD), aniridia, and cataract. The patient also had multiple other congenital anomalies and severe developmental delay. To explain his combination of anomalies, molecular genetic analysis from peripheral blood was performed in late 2018 and early 2019. Following normal findings with a panel diagnostic of 18 genes associated with congenital glaucoma, whole exome sequencing was performed and revealed a novel likely pathogenic heterozygous variant c.251G>T, p.(Gly84Val) in the *SOX11* gene (SRY-related HMG-box gene 11). The variant had occurred de novo. Thus, the multiple congenital anomalies and developmental delay of the patient represented Coffin-Siris syndrome 9 (CSS9, OMIM #615866).

**Conclusions:**

When eye diseases occur in combination with other systemic features, genetic analysis can be seminal. Results indicate that glaucoma is an occasional feature of patients with Coffin-Siris syndrome. As early treatment may improve the visual outcome of patients with glaucoma, we suggest that patients with Coffin-Siris syndrome should receive specific ophthalmological screening.

**Supplementary Information:**

The online version contains supplementary material available at 10.1186/s12886-020-01788-0.

## Background

Secondary childhood glaucoma develops as a result of reduced aqueous outflow due to a congenital or acquired ocular or systemic disease. Anterior segment dysgenesis (ASD) is a frequent feature of secondary childhood glaucoma [1] and includes the subtypes Peters anomaly, aniridia, and Axenfeld-Rieger malformation [2].

The Coffin-Siris syndromes (CSS) are a genetically heterogeneous group of congenital disorders characterized by intellectual disability, coarse facial features, hypoplastic or absent fifth fingernails or toenails, and multiple other anomalies including ocular features [3]. The various forms of Coffin-Siris syndrome have been shown to be caused by mutations in 11 different genes encoding subunits of a chromatin remodelling factor, the SWI/SNF (SWItch/Sucrose Non-Fermentable) complex.

This is the first report of secondary childhood glaucoma in Coffin-Siris syndrome. Molecular analysis revealed a likely pathogenic de novo heterozygous variant in the *SOX11* gene, indicating a diagnosis of Coffin-Siris syndrome 9 (CSS9). The glaucoma was caused by anterior segment dysgenesis of Peters type iridocorneal dysgenesis and aniridia.

## Case presentation

A 2 year old boy of Romanian descent was referred to the childhood glaucoma centre of the University Medical Centre of the Johannes Gutenberg- University, Mainz, Germany, for further evaluation and treatment. His mother had noticed corneal opacity of his left eye soon after birth and consulted ophthalmologists elsewhere. At the age of 1 year the boy’s left eye had been treated by cyclophotocoagulation due to constantly elevated intraocular pressure (IOP) with a maximum IOP of 43 mmHg (T_max_). Both eyes were treated with a topical hypotensive medication containing a prostaglandin, a beta-blocker, and a carbonic anhydrase inhibitor.

During our first ophthalmologic examination at the age of 2 years and 8 months in June 2018 he frequently rubbed his eyes, showed Bell’s phenomenon and variable horizontal and vertical pendular nystagmus. Using his right eye, he could track movements and localize large objects at 10–15 cm distance. Direct fixation of the objects was only possible when adding an acoustic signal. The right eye also was photophobic. His left eye showed no light perception. Three months later, further exploration under general anaesthesia revealed the following signs on the right eye: microcornea, a small central corneal opacity, mild conjunctival vascularization of the cornea, central and paracentral corneal thinning, aniridia with small remnants of iris at the temporal side, cataract, and normal fundus reflex (Additional file Fig. 1A-C). Using a hand-held Goldmann applanation tonometer (GAT) IOP was 23 mmHg (mean value is 16.22 mmHg, 95% CI 15.48–16.97) [4]. It is well described that during general anaesthesia IOP levels may be artificially lowered due to the sedation drugs [5]. IOP above 21 mmHg is considered to be elevated [6]. Corneal diameters were 10 mm horizontal and 6 mm vertical (mean value is 11.71 mm ± 0.42 mm) [7]. Axial length was normal at 21.68 mm (mean value of normal 2–72 months old children without ophthalmological symptoms is 20.97 mm ± 1.48 mm) [8]. His left eye showed microcornea, buphthalmos, severe corneal vascularization, absence of clear limbus borders, cataract, and complete corneal opacity (Additional file Fig. 1D). IOP was 20 mmHg (GAT) and axial length was elevated at 22.98 mm. Corneal diameters were 9 mm horizontal and 4 mm vertical. Gonioscopy was not possible due to corneal opacities in both eyes. Using ultrasonography, the anterior chamber of both eyes was abnormal with iridocorneal peripheral and central synechiae. Pachymetry was 341 μm (RE) and 382 μm (LE), indicating extreme corneal thinning (mean value of normal children without glaucoma is 551 μm) [9] as shown in the slit lamp image (Additional file Fig. 1C). A thin central cornea can result in erroneously low IOP when measuring with the Goldmann applanation tonometer [6]. Presumably the patient’s actual IOP was even higher than the measured values due to the corneal thinning and the pressure lowering effect of the anaesthesia. Four months later in January 2019, another exploration of both eyes under general anaesthesia showed fully attached retinae and normal vitreous bodies (Additional file Fig. 2A and B).

Upon clinical genetic examination at the age of 3 years and 4 months, his height was 98 cm (34th percentile, − 0.41 SD), weight was 13 kg (9th percentile, − 1.33 SD) and occipitofrontal circumference was 47 cm (microcephaly, < 1st percentile, − 3.11 SD). He had a coarse facies showing microcephaly, sparse hair, arched eyebrows, depressed nasal bridge, short nose, anteverted nostrils, short philtrum, prominent upper lip, macrostomia, prominent and everted lower lip, open mouth appearance, and low-set ears (Additional file Fig. 3A and B). He also had abnormal fifth fingers showing clinodactyly and hypoplastic terminal phalanges (Additional file Fig. 3C), cryptorchidism, and severe developmental delay. He had achieved sitting alone at the age of 2 years and 10 months and was able to stand only with extensive support.

Molecular genetic analysis was performed using peripheral blood as described previously [10]. Following normal findings using sequence analysis of 18 genes known to be associated with congenital glaucoma, we applied whole exome sequencing and identified a novel de novo heterozygous variant c.251G>T, p.(Gly84Val) in the SRY-related HMG-box gene 11 (*SOX11*). The variant had neither been described in the literature nor annotated in the ClinVar database and was absent in the parents and the general population. It alters a conserved codon and was predicted to be likely pathogenic by in silico prediction tools (MutationTaster, CADD, SIFT, Varsome).

Our medical and surgical treatment concentrated on the child’s right eye, considering it to be the only eye being able to perceive light. We first performed a limbal cell transplantation combined with amniotic membrane transplantation at the age of 3 years and 1 month, followed by an allogenic simple limbal epithelial transplantation (AlloSlet) and conjunctival covering. Thereafter we stopped topical hypotensive medication on the right eye. On the left eye the use of topical carbonic anhydrase inhibitor was continued. Unfortunately, when last seen at the age of 3 years and 7 months the boy did not show any improvement in his visual ability compared to before the operations.

## Discussion and conclusion

Upon ophthalmological evaluation the patient was diagnosed with secondary childhood glaucoma caused by ocular anterior segment dysgenesis (ASD) of Peters type iridocorneal dysgenesis in combination with congenital limbal stem cell deficiency (LSCD). The right eye showed congenital aniridia and cataract, the left eye a mature cataract. The complex ophthalmological features of the boy hindered exact phenotyping, which made us compare his signs and symptoms with features of two different types of ASD: aniridia and Peters anomaly (Additional file Table 1).

By ophthalmologic evaluation, aniridia was the most prominent sign. Congenital aniridia is characterized by “partial or near total absence of iris”, aniridia-associated keratopathy (AAK), glaucoma, cataract, foveal hypoplasia, optic disk hypoplasia, and nystagmus [11, 12]. Limbal stem cell deficiency promotes the development of AAK that features “thickening and vascularization of the peripheral cornea” resulting in corneal opacities and increased central corneal thickness. Aniridia can be associated with other abnormalities, for example in WAGR syndrome [11], and can be caused by genetic variants in *PAX6, FOXC1, PITX2, CYP1B1, FOXD3*, and *TRIM44*, respectively [12].

Peters anomaly (PA) is characterized by congenital central corneal opacity, posterior corneal defects, and central iridocorneal synechiae, and is often accompanied by “microcornea, anterior polar cataract, glaucoma [and] maldevelopment of the hyaloid system” [13]. PA may also include systemic features, such as craniofacial defects, central nervous system anomalies (e.g. developmental delay), skeletal defects (e.g. clinodactyly), congenital heart disease, and renal, genital or other anomalies”, and mutations have been reported in the *PAX6, PITX2, PITX3, FOXC1, FOXE3, CYP1B1, B3GLCT, COL4A, TFAP2A, HCCS, NDP, FLNA,* and *SLC4A11* genes [14].

The first two patients with Coffin-Siris syndrome caused by de novo *SOX11* mutations were described in 2014 [15]. Excluding our patient, only 14 patients with *SOX11* deletion or de novo mutation have been reported so far. Mutations compromised 8 loss-of-function variants including 7 gross deletions [16: cases 1–7] and a nonsense mutation [16: case 10] and 6 de novo missense variants [15: two cases; 16: cases 8 and 9 [16] one case; 3: one case]. Hempel et al. identified 10 new CSS9 patients and described their neurodevelopmental disorder and various skeletal findings assembling the features of CSS. Out of these, 9 (90%) showed 5th finger clinodactyly, 4 (40%) had hypoplasia of the 5th toenail, and 6 (60%) featured eye findings including hypermetropia (30%), oculomotor apraxia (20%) and notably, unilateral microphthalmia (10%) [17].

In an experiment with homozygous *Sox11*-deficient mice (Sox11^−/−^) Wurm, Sock et al. identified specific ophthalmological symptoms overlapping with the ocular manifestations in this patient. Their findings included microphthalmia in all cases, anterior coloboma in 82% of Sox11^−/−^ mice whose eyes were sectioned in the coronal plane, and Peters anomaly - which is a subtype of ocular anterior segment dysgenesis (ASD) - in 96% of Sox11^−/−^ mice whose eyes were investigated by histology. The authors concluded *SOX11* is required for “separation of the lens vesicle from the surface ectoderm and the closure of the anterior optic fissure”. While investigating the signalling pathways of *SOX11* the authors found that “a lack of bone morphogenetic protein 7 (BMP7) signalling might be causatively involved in the formation of the ocular phenotype of Sox11^-/-^ mice”. They suggested a similar pathogenesis in the development of ASD in humans [2].

Patients with CSS may also show hypophoria, astigmatism, strabismus, buphthalmos, nystagmus, microcornea, and cataract [18]. Some of these features (buphthalmos, nystagmus, microcornea, and cataract) correlate with symptoms of the boy we described. Further, microphthalmia and retinal dystrophy have been reported in individuals with CSS4 due to *SMARCA4* (SWI/SNF-Related, Matrix-Associated, Actin-Dependent Regulator of Chromatin, Subfamily A, Member 4) mutation [19, 20]. Very recently, the combination of open-angle glaucoma with CSS by *ARID1B* (AT-rich interaction domain-containing protein 1B) gene mutation was observed in two children diagnosed with infantile glaucoma due to high IOP [21].

The extraopthalmic clinical signs of our patient unequivocally represented a Coffin-Siris syndrome, here, Coffin-Siris syndrome-9 caused by a de novo heterozygous *SOX11* gene mutation. There have been only two reports of microphthalmia [16: case 1; 19: one case] and no report of aniridia or Peters anomaly in humans with CSS so far. However, the ocular phenotype in our patient was much more severe than in previously reported patients with CSS. Reasons for this are unknown. Unidentified modifier genes could be discussed, but we would propose variable clinical expression, or possibly a combination of both. Nevertheless, the ocular findings (of anterior segment dysgenesis, glaucoma, buphthalmos, nystagmus, microcornea, cataract) in this patient most likely do not represent a chance coincidence, because they resembled the findings in Sox11^−/−^ mice, which very frequently displayed microphthalmia, anterior coloboma, and Peters anomaly [2].

This is the first report of secondary glaucoma in a patient with Coffin-Siris syndrome 9 (caused by a de novo *SOX11* gene mutation) and the first report of secondary glaucoma with any form of Coffin-Siris syndrome. The findings of anterior segment dysgenesis in homozygous *Sox11*-deficient mice may be a hypothesis to understand the combined symptoms of Peters anomaly and aniridia in this patient [2]. Our findings confirm that molecular genetic analyses can help to clarify eye diseases that cannot be diagnosed by phenotyping alone, and suggest that patients with Coffin-Siris syndrome 9 should receive early and extensive ophthalmological evaluation.

## Supplementary Information

**Additional file 1.** Exploration of the eyes under general anaesthesia at the age of 2 years and 11 months. Image A: Right eye - microcornea, small central corneal opacity (marked with an arrow), and mild conjunctival vasculation of the cornea. Image B: Right eye - aniridia, clear lens, and normal red fundus reflex. Image C: Right eye – slit lamp image showing central (marked with an arrow) and paracentral corneal thinning. Image D: Left eye – buphthalmos, severe corneal vascularization

**Additional file 2.** Ocular ultrasound. Exploration of both eyes under general anaesthesia showed fully attached retinae and normal vitreous bodies. Image A: Right eye. Image B: Left eye.

**Additional file 3.** Clinical features of the patient. Image A: Frontal view at the age of 4 months. Note microcephaly, sparse hair, arched eyebrows, abnormal eyes, depressed nasal bridge, short nose, and low-set ears. Image B: Full view aged 1 year and 8 months. Note microcephaly, sparse hair, arched eyebrows, abnormal eyes, depressed nasal bridge, short nose, anteverted nostrils, low-set ears, generalized muscular hypotonia, and global retardation. Image C: Hands aged 3 years. Note alterations (hypoplastic terminal phalanges, clinodactyly) of fifth digits.

**Additional file 4.** Comparison of clinical features in aniridia, Peters anomaly, and this patient. ✓ = present. n.k. = not known due to corneal opacity.

## Data Availability

The datasets analysed in this study are not publicly available due to protection of medical data privacy but are available from the corresponding author on reasonable request.

## Abbreviations

AAK: Aniridia-associated keratopathy
ARID1B: AT-rich interaction domain-containing protein 1B
ASD: Anterior segment dysgenesis
BMP7: Bone morphogenetic protein 7
CSS: Coffin-Siris syndrome
GAT: Goldmann applanation tonometry
IOP: Intraocular pressure
LE: Left eye
LSCD: Limbal stem cell deficiency
OMIM: Online Mendelian Inheritance in Man
PA: Peters anomaly
RE: Right eye
SMARCA4: SWI/SNF-Related, Matrix-Associated, Actin-Dependent Regulator of Chromatin, Subfamily A, Member 4
SOX11: SRY-related HMG-box gene 11
SWI/SNF: SWItch/Sucrose Non-Fermentable

## References

[1] Papadopoulos M, Cable N, Rahi J, Khaw PT, B. I. G. eye study investigators (2007). The British infantile and childhood Glaucoma (BIG) eye study. Invest Ophthalmol Vis Sci.

[2] Wurm A, Sock E, Fuchshofer R, Wegner M, Tamm ER (2008). Anterior segment dysgenesis in the eyes of mice deficient for the high-mobility-group transcription factor Sox11. Exp Eye Res.

[3] Sekiguchi F, Tsurusaki Y, Okamoto N (2019). Genetic abnormalities in a large cohort of coffin-Siris syndrome patients. J Hum Genet.

[4] Farvardin M, Heidary F, Sayehmiri K, Gharebaghi R, Jabbarvand BM (2017). A comprehensive meta-analysis on intra ocular pressure and central corneal thickness in healthy children. J Public Health.

[5] European Glaucoma Society Terminology and Guidelines for Glaucoma (2017). 4th edition - chapter 2: classification and terminology supported by the EGS Foundation. Br J Ophthalmol.

[6] European Glaucoma Society Terminology and Guidelines for Glaucoma (2017). 4th edition - part 1 supported by the EGS Foundation. Br J Ophthalmol.

[7] Rufer F, Schroder A, Erb C (2005). White-to-white corneal diameter: normal values in healthy humans obtained with the Orbscan II topography system. Cornea.

[8] Sampaolesi R, Caruso R (1982). Ocular echometry in the diagnosis of congenital glaucoma. Arch Ophthalmol.

[9] Paletta Guedes RA, Pena AB, Paletta Guedes VM, Chaoubah A (2016). Longitudinal evaluation of central corneal thickness in congenital glaucoma. J Fr Ophtalmol.

[10] Kahrizi K, Huber M, Galetzka D (2019). Homozygous variants in the gene SCAPER cause syndromic intellectual disability. Am J Med Genet A.

[11] Lee H, Khan R, O'Keefe M (2008). Aniridia: current pathology and management. Acta Ophthalmol.

[12] Samant M, Chauhan BK, Lathrop KL, Nischal KK (2016). Congenital aniridia: etiology, manifestations and management. Expert Rev Ophthalmol.

[13] van Schooneveld MJ, Delleman JW, Beemer FA, Bleeker-Wagemakers EM (1984). Peters'-plus: a new syndrome. Ophthalmic Paediatr Genet.

[14] Weh E, Reis LM, Happ HC (2014). Whole exome sequence analysis of Peters anomaly. Hum Genet.

[15] Tsurusaki Y, Koshimizu E, Ohashi H (2014). De novo SOX11 mutations cause coffin-Siris syndrome. Nat Commun.

[16] Okamoto N, Ehara E, Tsurusaki Y, Miyake N, Matsumoto N (2018). Coffin-Siris syndrome and cardiac anomaly with a novel SOX11 mutation. Congenit Anom (Kyoto).

[17] Hempel A, Pagnamenta AT, Blyth M (2016). Deletions and de novo mutations of SOX11 are associated with a neurodevelopmental disorder with features of coffin-Siris syndrome. J Med Genet.

[18] Pallotta R (1985). Ocular anomalies in coffin-Siris syndrome. Ophthalmic Paediatr Genet.

[19] Errichiello E, Mustafa N, Vetro A (2017). SMARCA4 inactivating mutations cause concomitant coffin-Siris syndrome, microphthalmia and small-cell carcinoma of the ovary hypercalcaemic type. J Pathol.

[20] Cappuccio G, Brunetti-Pierri R, Torella A (2019). Retinal dystrophy in an individual carrying a de novo missense variant of SMARCA4. Mol Genet Genomic Med.

[21] Dolaghan MJ, George S, McLoone E (2019). Raised intra-ocular pressure in the setting of coffin-Siris syndrome. Eye (Lond).

