# Correction to: First observation of secondary childhood glaucoma in Coffin-Siris syndrome: a case report and literature review

**DOI:** 10.1186/s12886-021-01821-w

**Published:** 2021-01-22

**Authors:** Heidi Diel, Can Ding, Franz Grehn, Panagiotis Chronopoulos, Oliver Bartsch, Esther M. Hoffmann

**Affiliations:** 1 Department of Ophthalmology, University Medical Centre of the Johannes Gutenberg University Mainz, Langenbeckstr 1, D –, 55131 Mainz, Germany; 2 Institute of Human Genetics, University Medical Centre of the Johannes Gutenberg University Mainz, Mainz, Germany

**Correction to: BMC Ophthalmol 21, 28 (2021)**

**https://doi.org/10.1186/s12886-020-01788-0**

Following publication of the original article [1], we were notified that:

-Reference 16 and reference 17 have been interchanged. Reference 16 should be Hempel A et al. and reference 17 should be Okamoto N et al.

-The links to Additional files 2 to 4 were incorrect, all pointing towards Additional file 1.

The original paper has been corrected.
