# Supplementary figures and images for: First observation of secondary childhood glaucoma in Coffin-Siris syndrome: a case report and literature review
Source: BMC Ophthalmol. 2021 Jan 11;21:28. doi: 10.1186/s12886-020-01788-0 (PMC7802219; doi:10.1186/s12886-020-01788-0)

## Slide 1
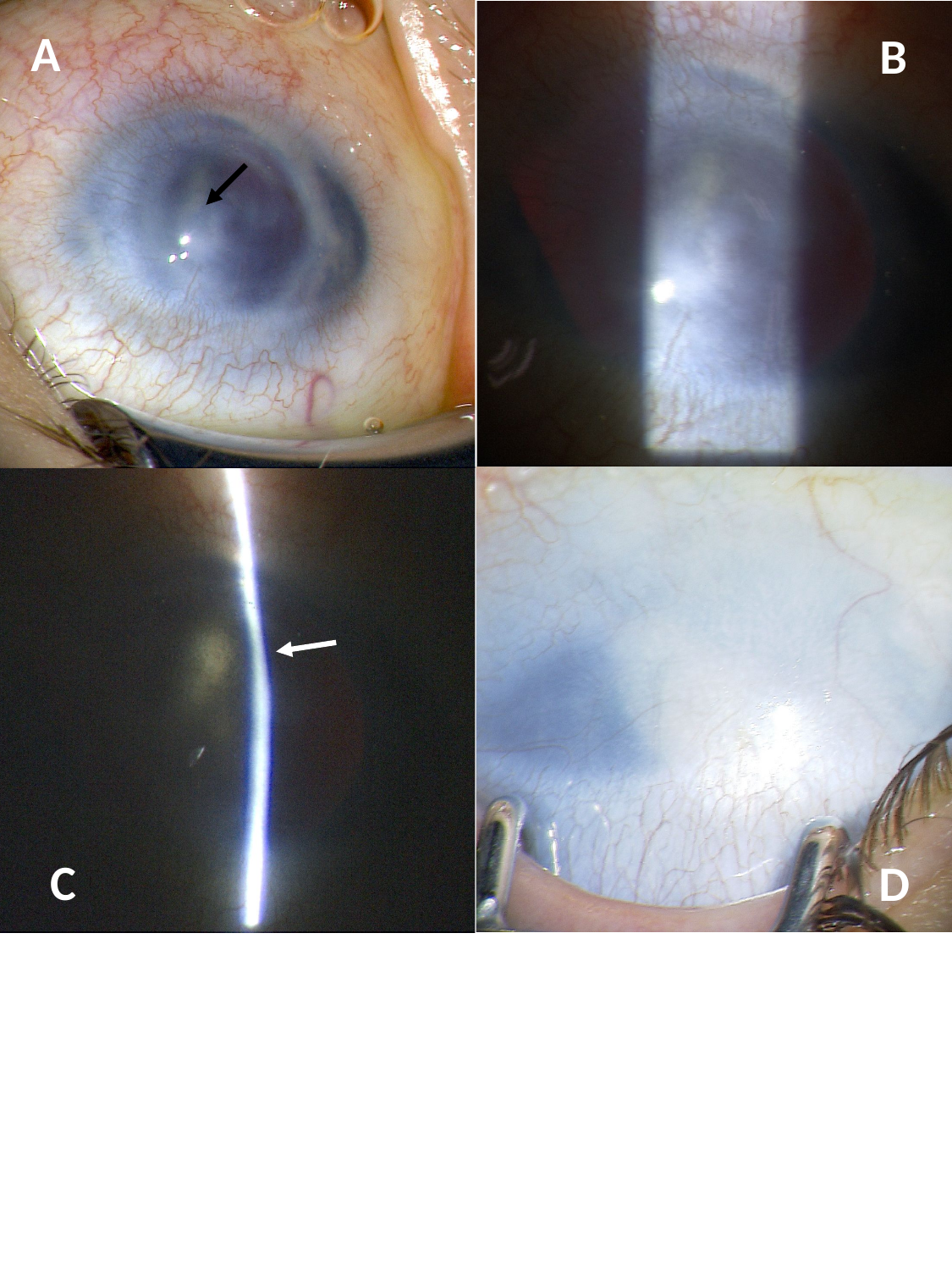

A
B
B
A
C
D

Supplement: Supplementary file 1 — Additional file 1. Exploration of the eyes under general anaesthesia at the age of 2 years and 11 months. Image A: Right eye - microcornea, small central corneal opacity (marked with an arrow), and mild conjunctival vasculation of the cornea. Image B: Right eye - aniridia, clear lens, and normal red fundus reflex. Image C: Right eye – slit lamp image showing central (marked with an arrow) and paracentral corneal thinning. Image D: Left eye – buphthalmos, severe corneal vascularization [file 12886_2020_1788_MOESM1_ESM.ppt]

## Slide 1
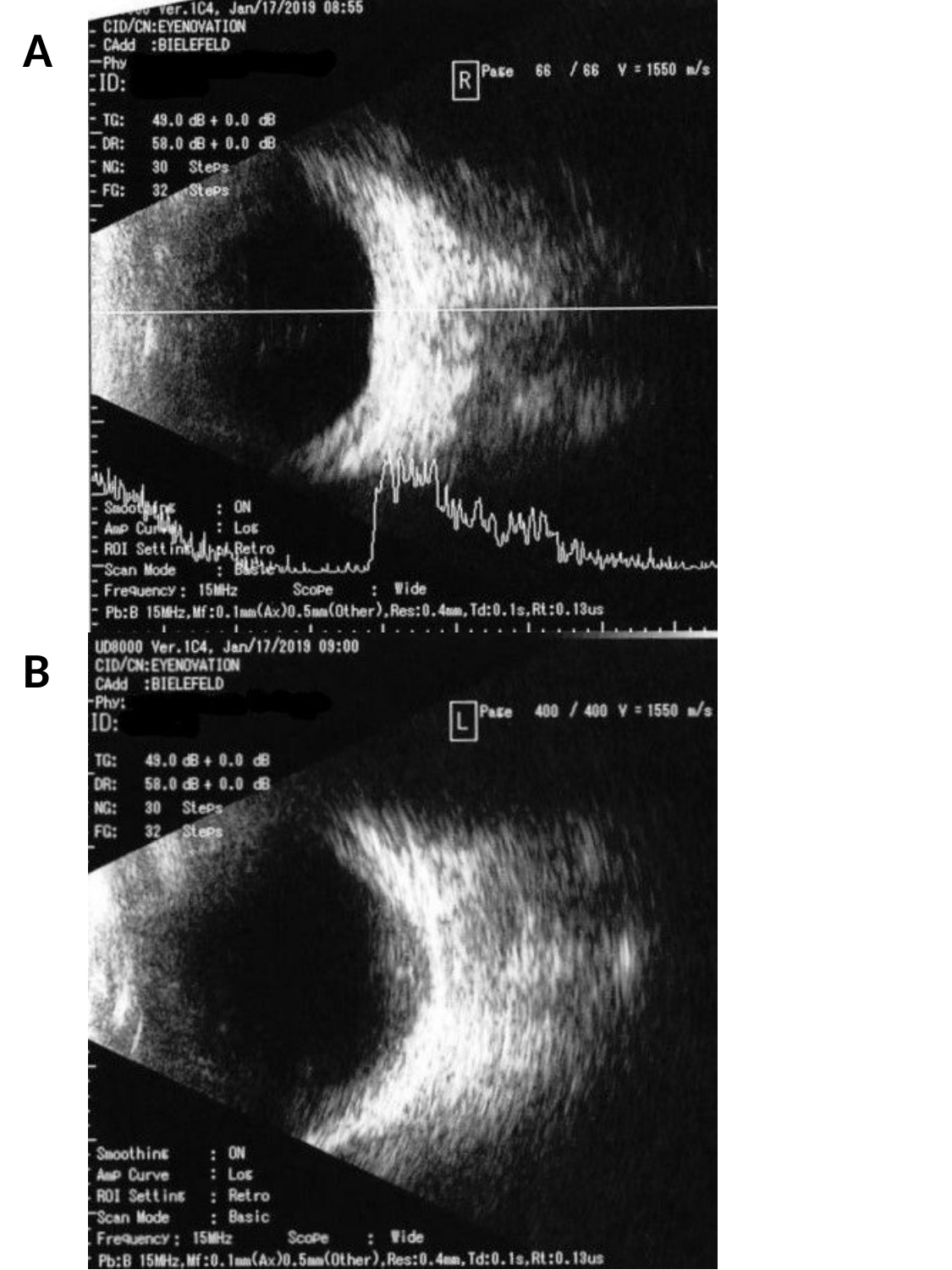

A
B

Supplement: Supplementary file 2 — Additional file 2. Ocular ultrasound. Exploration of both eyes under general anaesthesia showed fully attached retinae and normal vitreous bodies. Image A: Right eye. Image B: Left eye. [file 12886_2020_1788_MOESM2_ESM.ppt]

## Slide 1
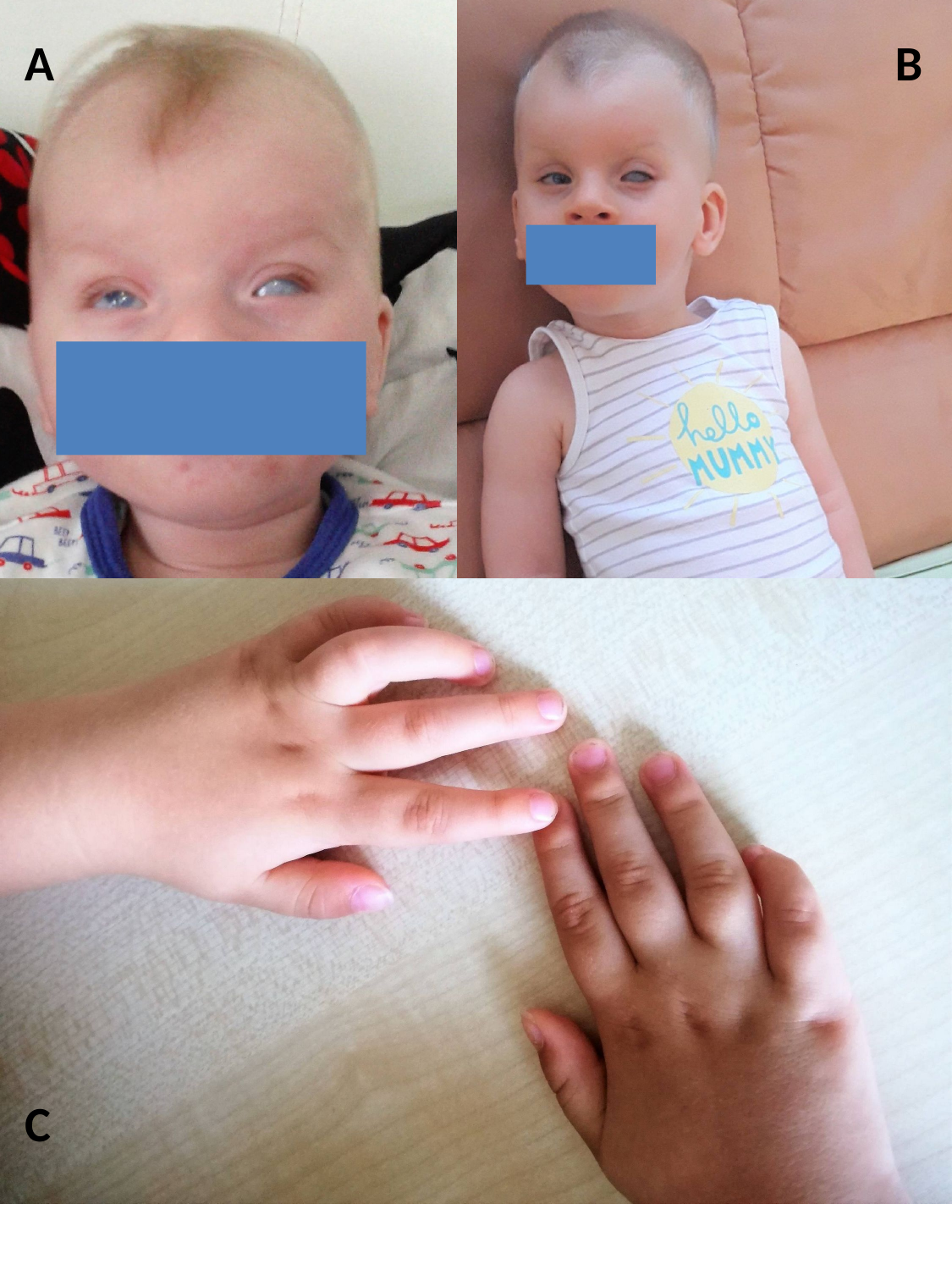

B
A
C

Supplement: Supplementary file 3 — Additional file 3. Clinical features of the patient. Image A: Frontal view at the age of 4 months. Note microcephaly, sparse hair, arched eyebrows, abnormal eyes, depressed nasal bridge, short nose, and low-set ears. Image B: Full view aged 1 year and 8 months. Note microcephaly, sparse hair, arched eyebrows, abnormal eyes, depressed nasal bridge, short nose, anteverted nostrils, low-set ears, generalized muscular hypotonia, and global retardation. Image C: Hands aged 3 years. Note alterations (hypoplastic terminal phalanges, clinodactyly) of fifth digits. [file 12886_2020_1788_MOESM3_ESM.ppt]
